# Impacts of Blended *Bombyx mori* Silk Fibroin and Recombinant Spider Silk Fibroin Hydrogels on Cell Growth

**DOI:** 10.3390/polym13234182

**Published:** 2021-11-29

**Authors:** Chavee Laomeephol, Apichai Vasuratna, Juthamas Ratanavaraporn, Sorada Kanokpanont, Jittima Amie Luckanagul, Martin Humenik, Thomas Scheibel, Siriporn Damrongsakkul

**Affiliations:** 1 Biomaterial Engineering for Medical and Health Research Unit, Faculty of Engineering, Chulalongkorn University, Bangkok 10330, Thailand; papomchavee@gmail.com (C.L.); Juthamas.R@chula.ac.th (J.R.); sorada.k@chula.ac.th (S.K.); jittima.l@pharm.chula.ac.th (J.A.L.); 2 Department of Obstetrics and Gynecology, Faculty of Medicine, Chulalongkorn University, Bangkok 10330, Thailand; apichai.v@chula.ac.th; 3 Biomedical Engineering Research Center, Faculty of Engineering, Chulalongkorn University, Bangkok 10330, Thailand; 4 Biomedical Engineering Program, Faculty of Engineering, Chulalongkorn University, Bangkok 10330, Thailand; 5 Department of Chemical Engineering, Faculty of Engineering, Chulalongkorn University, Bangkok 10330, Thailand; 6 Department of Pharmaceutics and Industrial Pharmacy, Faculty of Pharmaceutical Sciences, Chulalongkorn University, Bangkok 10330, Thailand; 7 Department of Biomaterials, Faculty of Engineering Science, University of Bayreuth, Prof.-Rüdiger-Bormann Str. 1, 95447 Bayreuth, Germany; martin.humenik@bm.uni-bayreuth.de

**Keywords:** silk fibroin, spider silk, hydrogel, self-assembly, cell culture

## Abstract

Binary-blended hydrogels fabricated from *Bombyx mori* silk fibroin (SF) and recombinant spider silk protein eADF4(C16) were developed and investigated concerning gelation and cellular interactions in vitro. With an increasing concentration of eADF4(C16), the gelation time of SF was shortened from typically one week to less than 48 h depending on the blending ratio. The biological tests with primary cells and two cell lines revealed that the cells cannot adhere and preferably formed cell aggregates on eADF4(C16) hydrogels, due to the polyanionic properties of eADF4(C16). Mixing SF in the blends ameliorated the cellular activities, as the proliferation of L929 fibroblasts and SaOS-2 osteoblast-like cells increased with an increase of SF content. The blended SF:eADF4(C16) hydrogels attained the advantages as well as overcame the limitations of each individual material, underlining the utilization of the hydrogels in several biomedical applications.

## 1. Introduction

Hydrogels are well suited as scaffolds for tissue engineering due to their characteristics resembling natural extracellular matrices. Hydrogels can be applied in various biomedical fields, such as injectable hydrogels or printable bioinks for space-filling or cell/biological factor delivery [1]. Silk fibroin is a naturally derived fibrous protein which is widely used as a base material in hydrogel fabrication, due to its self-assembly, mechanical stability of the gels, and biocompatibility [2]. In this work, silk fibroin derived from two different sources, *Bombyx mori* silk cocoons and recombinant spider dragline silk proteins, were chosen to form blended hydrogels, and their cytocompatibility was tested in vitro.

Combining two materials is an approach to gain the advantage from both materials as well as to overcome some limitations to achieve products with desired features [3]. Silk fibroin (SF) can be derived at high amounts from silkworms by isolating the SF solution from silk glands or dissolving silk cocoons with the drawback of some inhomogeneities common to all nature-derived materials. However, SF solution can be produced under certified conditions, and SF is already available from several companies, such as Fibrothelium GmbH, Aachen, Germany, Sigma-Aldrich, MO, USA, and Advanced Biomatrix, CA, USA. Glycine-alanine repeats of SF can form beta sheet structures, which is relevant for self-assembly as well as the physical strength of the obtained materials [4]. However, self-gelation of SF is extremely slow (ca. 7–12 days depending on the SF concentration) [5], which is impractical in various applications, especially for cell encapsulation. Several strategies have been applied to accelerate the self-assembly process of SF, including an application of physical or mechanical forces, an addition of chemicals, as well as a simple blending with other polymers. Regarding the works from Mandal BB’s group, non-mulberry SF, with its primary structure containing a high ratio of alanine-glycine and poly-alanine sequences, was simply blended with *B. mori* SF, and rapid gelation can be achieved. The biological properties were drastically improved due to the presence of arginine-glycine-aspartic acid (RGD) motifs in non-mulberry SF [6,7,8,9].

The recombinant spider silk protein (eADF4(C16)), derived from ADF4 of the dragline silk protein of *Araneus diadematus* containing poly-alanine sequence, can be spontaneously assembled into hydrogels within hours depending on the protein concentration or the ionic strength of the solution [10,11]. The advantage of this recombinant protein is its large availability with continuous properties. eADF4(C16) is a commercially available spider silk protein from AMSilk GmbH, Martinsried, Germany. Major limitation of eADF4(C16) is cell adhesion and proliferation unless the protein is genetically modified, e.g., with a tag comprising the cell adhesion motif RGD [12]. Hence, binary blending of SF and eADF4(C16) could expectedly be beneficial in enhancing the interaction with cells, as well as accelerating the gelation process within a range suitable for practical uses.

Herein, the gelation of SF and eADF4(C16) blends was evaluated. Since the hydrogels were proposed to serve as cell-loaded substrates, the interaction with primary cells and cell lines was tested. Physico-chemical properties of the hydrogels, which could affect the cellular behavior, namely micromorphology, hydrophobicity, and protein diffusivity, were also identified. This work as a proof-of-concept study provides information of blended SF:eADF4(C16) hydrogels for further applications e.g., cell-encapsulation for cell delivery or injectable or printable materials.

## 2. Materials and Methods

### 2.1. Material

Thai *Bombyx mori* silk cocoons were received from Queen Sirikit Sericulture Center, Nakhon Ratchasima province, Thailand. Recombinant protein eADF4(C16) based on dragline silk protein ADF-4 of *Araneus diadematus* was produced according to the published protocol [13]. Briefly, a bacterial expression plasmid containing gene corresponding to 16 repeats of module C of ADF4 protein (sequence: GSSAAAAAAAASGPGGYG PENQGPSGPGGYGPGGP) was induced in *E. coli* strain BLR(DE3) using isopropyl β-D-1-thiogalactopyranoside (IPTG). Approximately 3–4 h after the induction, cells were harvested, lysed and eADF4(C16) was isolated by precipitation using 30% ammonium sulfate, before redissolution in 6 M guanidine thiocyanate (GdnSCN) and lyophilization. All reagents used in this study were of analytical grade and supplied from Sigma-Aldrich, MO, USA, unless otherwise stated.

To extract SF from silk cocoons, 40 g of the cocoons were boiled in 1 L of 0.02 M Na_2_CO_3_ for 20 min twice to remove sericin, before leaving to dry. Four gram of dried silk fiber was dissolved in 16 mL of 9.3 M LiBr (1:4 weight-to-volume ratio), and incubated at 60 °C for 4 h. After that, LiBr was eliminated by dialyzing the SF solution against deionized water for 48 h using a dialysis tube with molecular weight cut-off (MWCO) of 12–16 kDa [14]. The concentration of the protein solution was determined from dry solid weight. To prepare the sterile SF solution, the solution was autoclaved at 121 °C for 20 min and stored in a refrigerator until usage.

eADF4(C16) powder was dissolved in 6 M GdnSCN at a concentration of 4 mg/mL and incubated at 37 °C for 1 h. The solution was then dialyzed against 10 mM Tris-HCl buffer (pH 7.4) using a dialysis tube with MWCO of 6–9 kDa. Subsequently, the protein solution was concentrated using dialysis against 20% *w*/*v* polyethylene glycol (PEG; M_n_ = 35 kDa), and the concentration was determined from the absorbance at 280 nm [10]. The eADF4(C16) was UV-irradiated for 20 min for sterilization.

### 2.2. Gelation Kinetics

Gelation of the protein solutions and blends was investigated using the change of turbidity, as the gelation is associated with fibril assembly causing light scattering [5,10]. 2% and 3% *w*/*v* SF and eADF4(C16) solutions were prepared and mixed at the volume ratio of SF:eADF4(C16) of 10:0, 7:3, 5:5, 3:7 and 0:10. The effects of different buffers, including Dulbecco′s Modified Eagle′s Medium (DMEM), phosphate buffer saline (PBS), and normal saline solution (NSS), on the gelation kinetics were investigated by supplementing the protein mixtures with 10X concentrated solutions. The samples were prepared from the sterile stock solutions using aseptic techniques to avoid microbial contamination. 100 µL of the mixtures were transferred to a 96-well plate, and the change of visible light absorption at 550 nm was measured using a microplate reader (FLUOstar Omega, BMG Labtech, Ortenberg, Germany). The temperature was controlled at 37 °C, and the microplate was sealed to prevent water evaporation. The measurement was conducted every 15 min for 40 h.

### 2.3. Visualization of Microstructures

The morphologies of hydrogels were observed upon freeze-drying using a field emission scanning electron microscope (FESEM; JSM-7610F, Jeol, Tokyo, Japan). The samples were prepared as above described by incubating the mixtures in tight-sealed vials at 37 °C until achieving the complete gelation. The samples were immediately frozen using liquid nitrogen for 30 min and at −80 °C overnight before lyophilization. Flash freezing the samples in liquid nitrogen was performed to preserve the microstructure of the hydrogels.

To visualize the micromorphological structure using FESEM, the freeze-dried samples were cut and coated with platinum. The FESEM was operated with an acceleration voltage of 5 kV.

### 2.4. Quantitative Determination of Secondary Structures

The structures of freeze-dried hydrogels were determined using Fourier-transform infrared (FTIR) spectroscopy in an attenuated total reflection (ATR) mode (Nicolet™ iS™ 5, Thermo Fisher Scientific, Waltham, MA, USA). The absorbance spectra within the range of 4000 to 800 cm^−1^ were collected with 1.0 cm^−1^ resolution. The secondary structures were quantified using Fourier self-deconvolution (FSD) and curve-fitting techniques according to the established protocol [15]. In brief, the deconvolution of amide I spectrum (1725–1575 cm^−1^) was obtained using Omnic 8.0 software (Thermo Fisher Scientific, Waltham, MA, USA) by fitting the Voigt line shape with a half-bandwidth of 25 cm^−1^ and an enhancement factor of 2.5. Subsequently, the deconvoluted spectrum was fitted with the Gaussian function using Origin Pro 9.0 software (OriginLab, MA, USA). The content of beta sheet structure was obtained from the peak area between 1616–1637 cm^−1^. Other structures, namely random coil, alpha-helix and beta turn, were determined from the peaks at 1638–1655, 1656–1662 and 1663–1696 cm^−1^, respectively [16].

### 2.5. Protein Adsorptivity of Blended Hydrogels

Fetal bovine serum (FBS), a mixture of soluble proteins which is widely used in in vitro biological experiments, was selected to study the protein adsorptivity of the SF:eADF4(C16) hydrogels. 2% Hydrogels were prepared in a silicone mold, cut into a disc shape with a diameter of 5 mm and a thickness of 1 mm, and immersed in 10% FBS in PBS buffer (pH 7.4) at 37 °C for a particular period. After that, the hydrogels were washed with PBS to remove redundant proteins and incubated in 1 mL of PBS at 4 °C overnight with gentle shaking to extract the protein. Supernatants obtained from the samples immersed in PBS overnight without FBS were used as blanks. Protein content in the extracted solution was determined using Bio-Rad protein assay (Bio-Rad Laboratories, Hercules, CA, USA) according to the manufacturer’s instruction. 80 µL of the supernatant was mixed with 20 µL of the dye, incubated at room temperature for 15 min, and the absorbance was measured at 595 nm. 

### 2.6. Cell Preparation

Three different cell types, including human adipose-derived stromal cells (hASCs), L929 mouse fibroblasts, and SaOS-2 human osteoblast-like cells, were chosen to investigate their activities while culturing on the developed SF:eADF4(C16) hydrogels. Primary hASCs were used as a model for tissue engineering applications. L929 and SaOS-2 were selected to represent normal and tumor cells, respectively.

Human ASCs were isolated from subcutaneous fat tissues collected from female participants enrolled to Chulalongkorn Memorial Hospital, Thailand for laparotomy with an approval from institutional ethic committee on human research, Faculty of Medicine, Chulalongkorn University (project no. 416/61). Isolation and culture procedures were conducted following established protocols [17,18]. Briefly, 10–15 g fat tissue was washed with PBS before enzymatically digested using 0.1% collagenase type II (Gibco, New York, NY, USA) supplemented with 1% bovine serum albumin at 37 °C with continuous shaking for 1 h. The digested specimen was then centrifuged, and the upper oil layer was removed. The bottom dark brown layer, known as stromal vascular fraction (SVF), was collected, resuspended with PBS and centrifuged. After that, SVF was resuspended with culture medium (DMEM/F12 + 10% FBS + 1% antibiotics) and transferred to a T-75 tissue culture flask. The culture was maintained at 37 °C with fed air supplemented with 5%CO_2_.

After initial plating for 48 h, cells were washed with PBS to remove unattached cells and refed with the new medium. Typically, hASCs reached 80–90% confluency within 2 weeks. Subculture was performed using TrypLE Express enzyme (Thermo Fisher Scientific, Waltham, MA, USA) according to the manufacturer’s advice with a subculture ratio of 1:2.

L929 and SaOS-2 were cultured in DMEM supplemented with 10% FBS and 1% antibiotics. The cells were maintained in a CO_2_-incubator, and the subculture was treated using trypsin (Thermo Fisher Scientific, Waltham, MA, USA) according to the manufacturer’s protocol.

### 2.7. Evaluation of Cell Attachment and Proliferation on the Silk Hydrogels

Hydrogels were prepared as mentioned above using autoclaved SF and UV-irradiated eADF4(C16) solutions, and the overall concentration of protein mixtures was fixed at 2% *w*/*v*. 100 µL of the protein mixtures were transferred to a tissue culture-treated 48-well pate and incubated at 37 °C under humidified atmosphere for at least 48 h to allow complete gelation. For pure SF solutions, gelation was accelerated using sonication at 40% amplitude for 30 s [19].

All hydrogels were hydrated with the complete media for 24 h prior to cell seeding. hASCs at a density of 5000 cell/cm^2^, L929 and SaOS-2 at a density of 10,000 cell/cm^2^ were seeded on the hydrogels. Seeding density was based on the proliferation profile of each cell, of which its logarithm growth phase should be achieved within 1 to 5 days. Cells cultured on the tissue culture-treated plate (TCP) were used as controls. The cell culture media, DMEM/F-12 + 10%FBS + 1% antibiotics and DMEM + 10%FBS + 1% antibiotics were used for culturing hASCs and L929 or SaOS-2, respectively, and the samples were stored in a CO_2_ incubator at 37 °C and 5% CO_2_ supplementation.

On day 1, 3, 5 and 7 after cell seeding, the cell proliferation was determined using the 3-(4,5-dimethylthiazol-2-yl)-2,5-diphenyltetrazolium bromide (MTT) assay (Thermo Fisher Scientific, Waltham, MA, USA) according to the manufacturer’s protocol. Briefly, cells were washed with PBS, treated with 0.5 mg/mL MTT solution, and incubated at 37 °C in the dark for 30 min. After that, the MTT dye was removed and replaced with dimethyl sulfoxide (DMSO) to extract the precipitated formazan. The blue solution was then retrieved, and its absorbance at 570 nm was measured with a visible-light background correction at 650 nm.

Cell morphology was observed using a phase contrast imaging as well as fluorescent live-dead staining with calcein AM and propidium iodide (PI) dyes (Thermo Fisher Scientific, Waltham, MA, USA). At the designated time-points, cells were washed and stained with the fluorescent dyes. Bright-field and fluorescent images were obtained using a fluorescence microscope (Nikon Eclipse 80i, Nikon, Tokyo, Japan) with green and red filters to visualize calcein AM stained (lived) and PI stained (dead) cells, respectively.

### 2.8. Statistical Analysis

Statistical analysis was performed using IBM^®^ SPSS^®^ Statistics software version 22 under the license of Chulalongkorn University. Data was analyzed using one-way analysis of variance (ANOVA) with Bonferroni post-hoc tests at the significant level of 0.05.

## 3. Results

### 3.1. Gelation Time of SF:eADF14(C16) Blends

**Figure 1:**
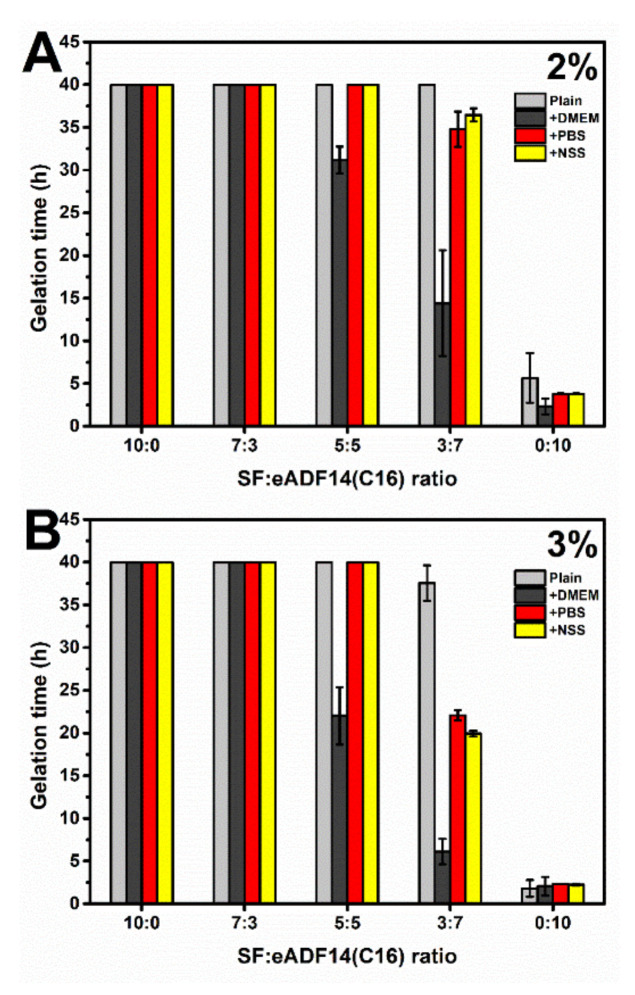
Gelation time of (**A**) 2% and (**B**) 3% *w*/*v* of SF:eADF4(C16) solutions with different ratios as indicated and upon addition of Dulbecco′s Modified Eagle′s Medium (DMEM), phosphate buffer saline (PBS) and normal saline solution (NSS) at 37 °C. “Plain” refers to SF:eADF4(C16) blends in the absence of salts. The gelation time was interpreted from the time-point at which the absorbance values reached the half-maxima. The experimental time was limited to 40 h. The ratio indicates the volume ratio of the respective 2% and 3% protein solutions.

The gelation of fibroin solutions was associated with the formation of heterogenous microstructures, which affected the light scattering degree in the visible range [5]. Therefore, the gelation can be noticed from the point at which an abrupt change of the visible-light absorbance value occurs. Figure 1 demonstrates the gelation time of 2% and 3% SF:eADF14(C16) blends. Only eADF4(C16) showed gel formation within less than a day, while a blending with SF significantly prolonged the gelation time. Increasing protein concentrations resulted in faster gelation. The SF:eADF4(C16) samples at a ratio 3:7 underwent the gelation in less than 40 h. Furthermore, a supplementation with DMEM, PBS, and NSS significantly reduced the gelation time, especially for blended samples (5:5 and 3:7 ratio). DMEM addition yielded slower gelation kinetics in case of blended 5:5 and 3:7 SF:eADF4(C16) solutions, when compared to those in presence of PBS and NSS.

### 3.2. Micromorphology of the Freeze-Dried Hydrogels

**Figure 2:**
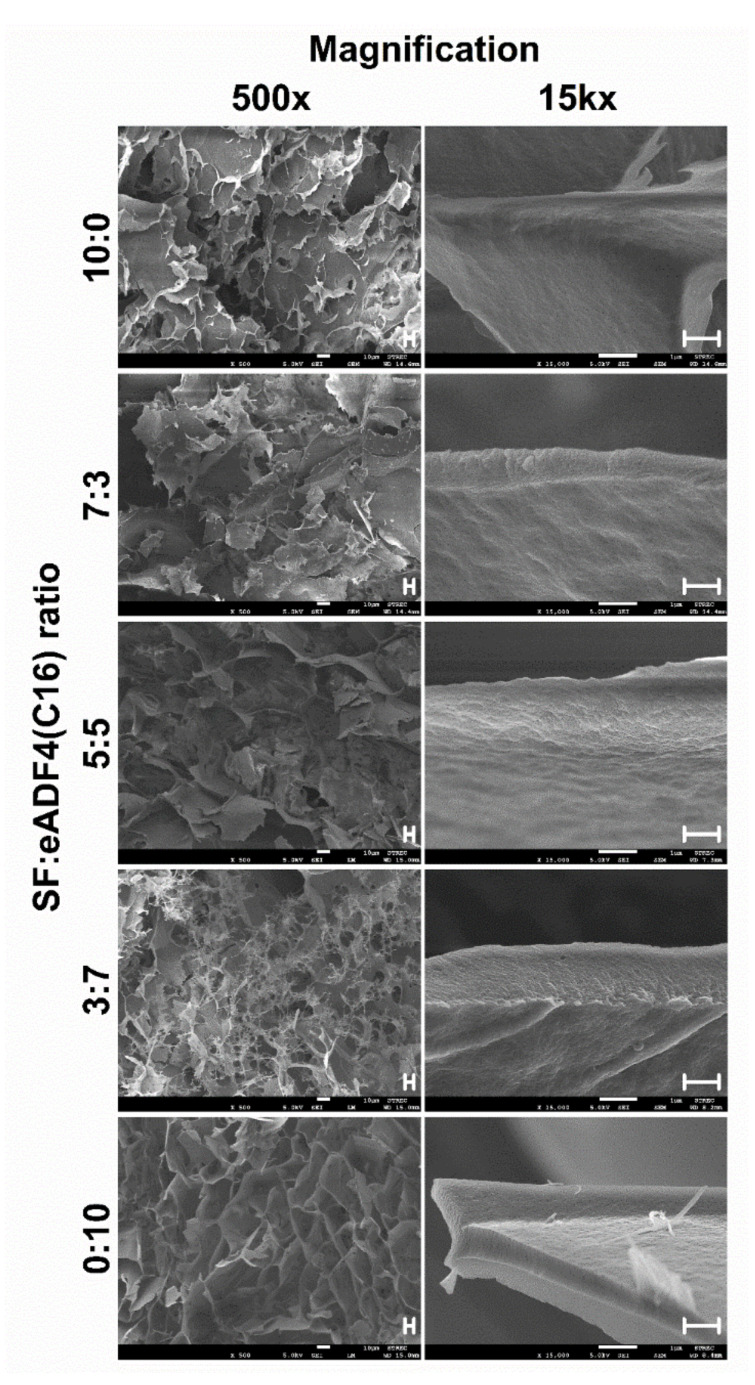
FESEM images of freeze-dried hydrogels. The number on the left indicates the SF:eADF4(C16) volume ratio. The scale bars of 500× and 15 kx magnification are 10 and 1 µm, respectively.

The microstructures of freeze-dried hydrogels were visualized using FESEM (Figure 2). The microstructures of all samples presented a high porosity with a ridge- or wall-like structure, and the fracture surfaces displayed an accumulation of nanofibers.

### 3.3. Secondary Structures of the Hydrogels

**Figure 3:**
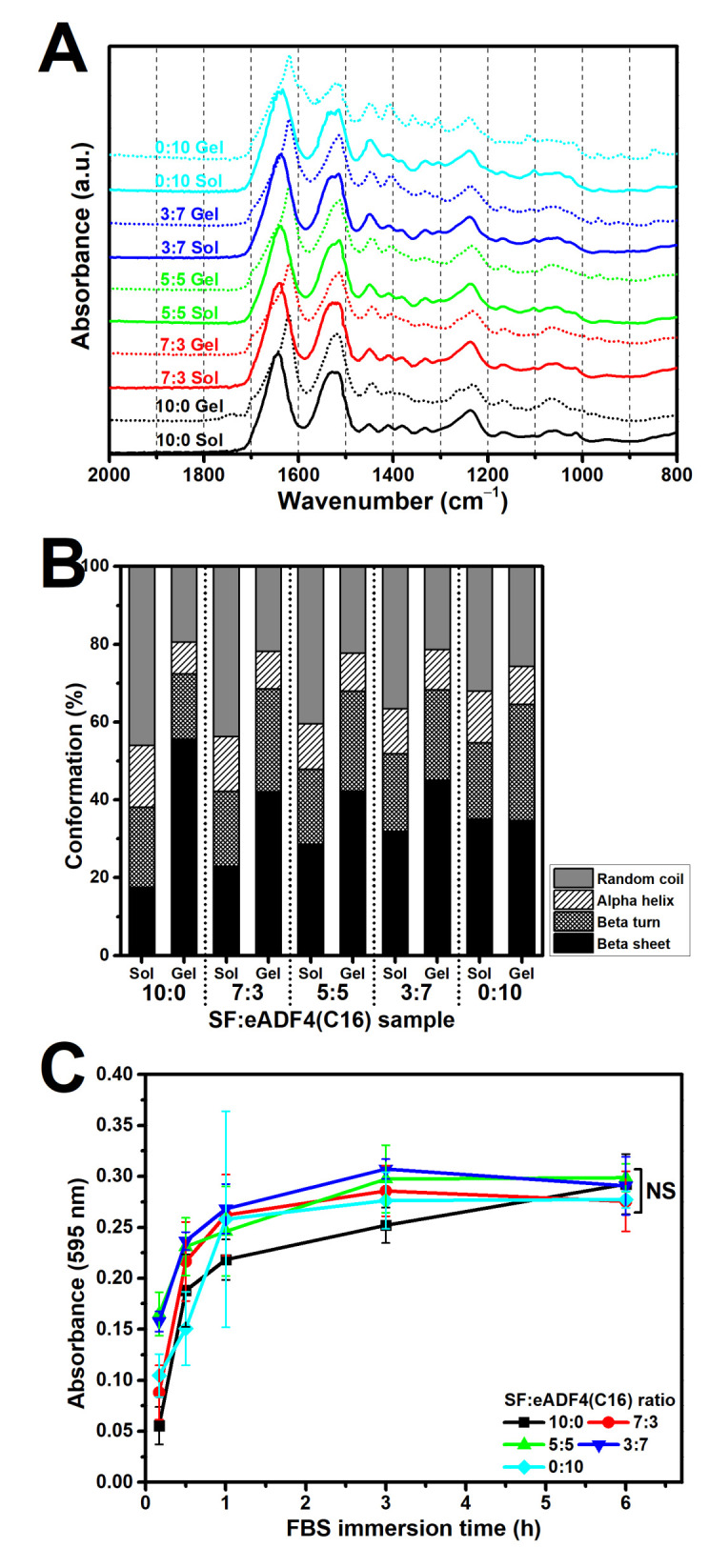
Physico-chemical properties of the SF:eADF4(C16). (**A**) FTIR spectra of freeze-dried hydrogels, (**B**) The content of protein conformation, quantified from the amide I region of FTIR spectra, and (**C**) Adsorption of proteins in the hydrogels after immersion in 10% FBS. NS: non-significant difference.

Figure 3A,B display the FTIR spectra and the secondary structure content of the freeze-dried SF:eADF4(C16) hydrogels, respectively. Comparing in sol and gel state, the peak shift from approximately 1650 cm^−1^ of the sol groups toward a lower wavenumber (1625 cm^−1^) of the gel group can be noticed, indicating a transition of the predominated random coil structure in the sol state to a beta sheet structure after gelation. The results were in accordance with the amount of secondary structures quantified using FSD and curve-fitting. A reduction in random coil and an increase of beta sheet structure could be clearly observed, especially in samples containing SF. The samples with higher eADF4(C16) content possessed a higher beta sheet and lower random coil content in the sol state, which slightly changed after gelation.

### 3.4. Protein Adsorptivity of the Hydrogels

Adsorption of soluble proteins in SF:eADF4(C16) hydrogels was determined using FBS as a model (Figure 3C). For all samples, FBS can be rapidly adsorbed onto the hydrogels in the first hour of immersion before maintaining a plateau. The adsorption of the proteins in the SF hydrogel (10:0) was slower than in the others, but the identical protein level could be achieved within 6 h.

### 3.5. Proliferation of Cells Cultured on the Silk Hydrogels

**Figure 4:**
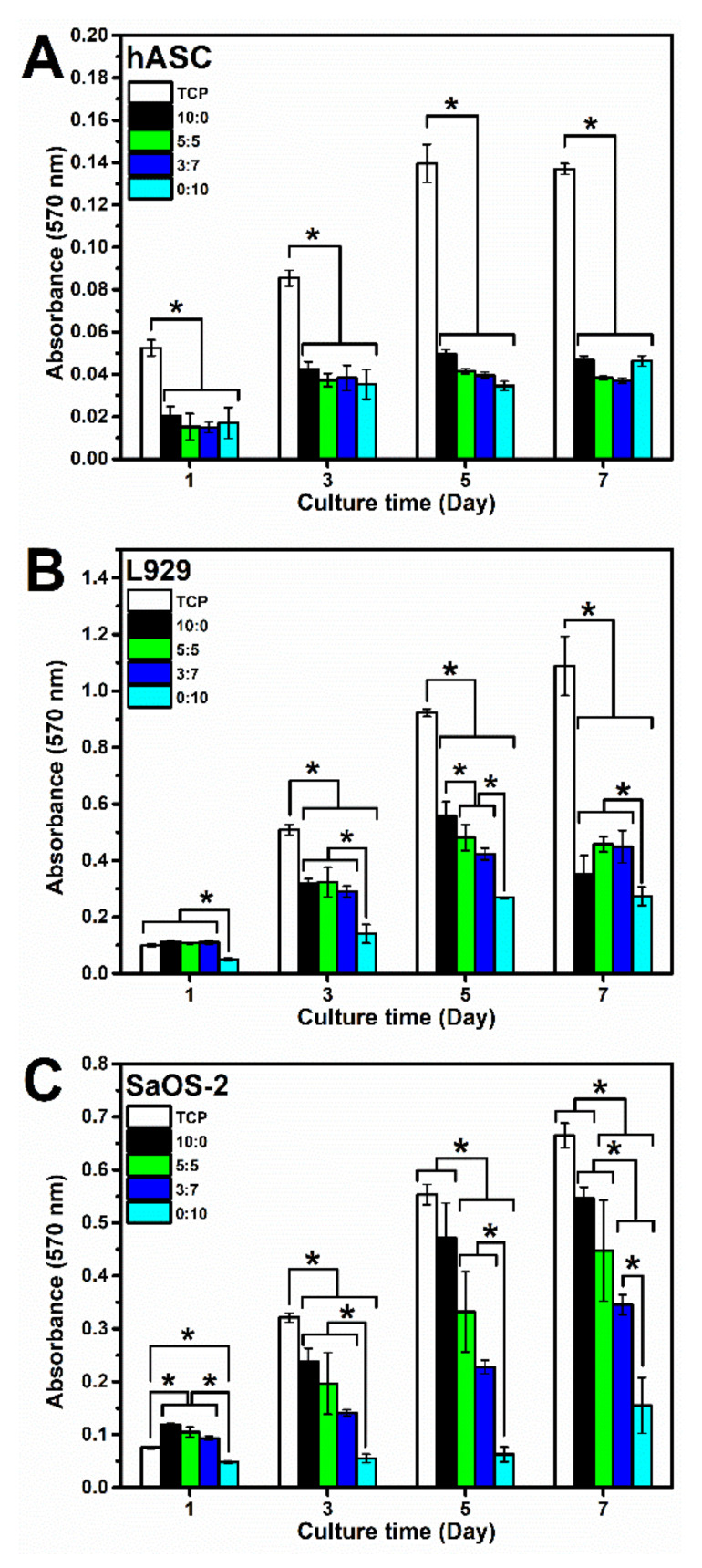
Cell proliferation determined using the MTT assay of (**A**) human adipose-derived stromal cells (hASC), (**B**) mouse L929 fibroblasts, and (**C**) human SaOS-2 osteosarcoma cells cultured on 2% SF:eADF4(C16) hydrogels for 7 days. Cells cultured on tissue culture. The asterisk (*) indicates the statistical difference at *p*-value ≤ 0.05.

Due to the very long gelation time, gelation of pure SF was accelerated using ultrasonication, and the 7:3 SF:eADF4(C16) hydrogel was omitted from the cell culture experiment. Cell proliferation was determined from MTT assay (Figure 4). For primary cells, such as hASC, the results showed no significant difference among all samples. Cells on TCP presented the growth phase in the first five days before plateauing on day 7 (population doubling time (PDT) = 61.3 h), while those on the silk hydrogels showed low proliferation rates after 3 days (Figure 4A). The proliferation of two cell lines, L929 and SaOS-2 was depended on the sample composition, as samples with higher SF content showed enhanced cellular activities. L929 cells (PDT on TCP = 21.0 h) cultured on hydrogels containing SF (10:0, 5:5, 3:7 SF:eADF4(C16)) showed a similar behavior, while the growth of cells on the eADF4(C16) (0:10) hydrogel was significantly lower (Figure 4B). Similar results were noticed for SaOS-2 (PDT on TCP = 25.9 h) (Figure 4C). The cells on 2% SF hydrogels presented the highest growth rate, which was not significantly different from those on TCP for all time-points. The absorbance values were lower depending on an increasing eADF4(C16) content, with the lowest cell activity for 2% eADF4(C16).

### 3.6. Morphology of Cells on the Hydrogels

**Figure 5:**
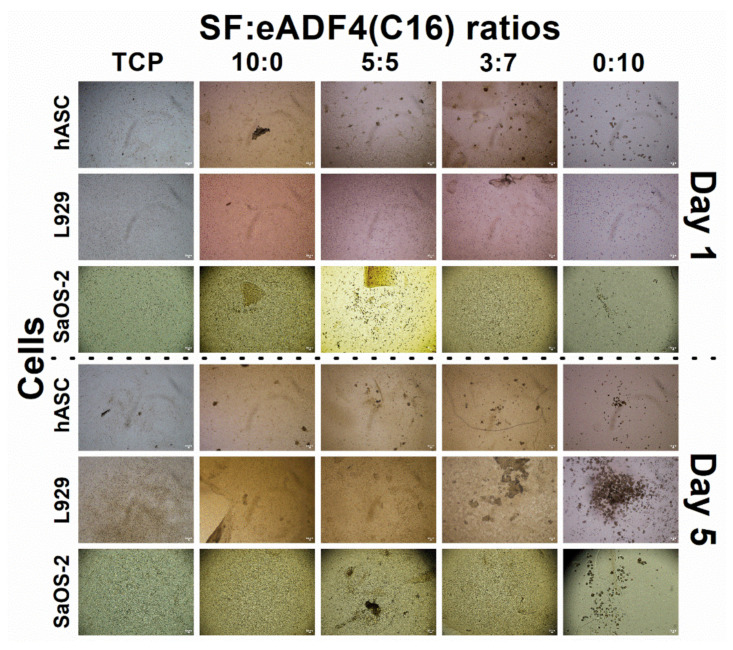
Bright-field images of hASC, L929 and SaOS-2 cells cultured on 2% SF:eADF4(C16) hydrogels on day 1 (**top** panel) and day 5 (**bottom** panel) (scale bar = 50 µm).

**Figure 6:**
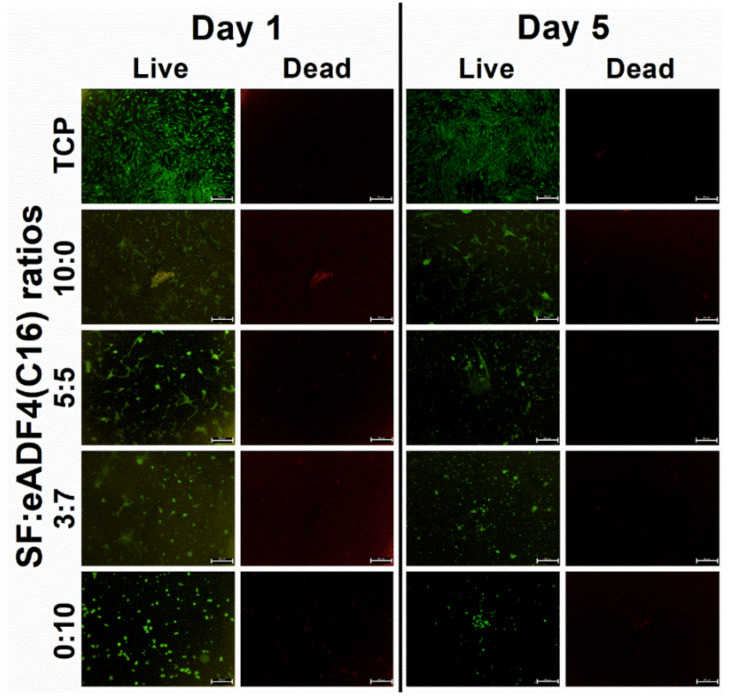
Fluorescence images of calcein AM (Live) and PI (Dead) stained hASC cells cultured on 2% SF:eADF4(C16) hydrogels on day 1 and day 5 (scale bar = 500 µm).

Bright-field images of hASC, L929, and SaOS-2 (Figure 5) cultured on silk hydrogels for 1 and 5 days showed the cell morphology after the initial attachment and in the exponential growth phase, respectively. Fluorescent images of hASCs (Figure 6) demonstrated live and dead cells, stained by calcein AM and PI dyes, respectively. The number of cells visualized from the images were in accordance with the cell proliferation results. It can be recognized that cells attached to hydrogels containing SF within 1 day after seeding. In contrast, cells on hydrogels with an increasing content of eADF4(C16) were less stretched and presented a lower attachment. On pure 2% eADF4(C16), cells could not attach well and preferably formed cell aggregates. 

## 4. Discussion

Blended SF:eADF4(C16) hydrogels were fabricated in order to combine the advantages of eADF4(C16) in facilitating rapid gelation and enhanced interactions with cells provided by SF. As shown in Figure 1, it can be clearly seen that the addition of eADF4(C16) can induce faster gelation of SF, i.e., the gelation of 5:5 and 3:7 hydrogels occurred within 48 h. The gelation of pure 2% and 3% SF solutions were not observed after one week, which was in agreement with a previous study [20]. As expected, an increase of protein concentration from 2% to 3% led to a shorter gelation time, due to a higher opportunity in chain-chain interactions [5,10]. Additionally, the presence of cations, especially the mixtures of monovalent and divalent cations as in DMEM, significantly shortened the gelation time of SF:eADF4(C16) blends. This finding confirmed the previous study in which the addition of DMEM and divalent cations, such as Ca^2+^, triggered a faster eADF4(C16) hydrogel formation [11]. Also, accelerated gelation of SF was achieved in presence of the divalent cation Ca^2+^, but not the monovalent K^+^ [21]. However, our results showed that mixtures of monovalent cations, such as in PBS, and NSS, were able to affect the gelation of SF:eADF4(C16). We propose that the presence of cations mostly influences assembly of eADF4(C16) rather than that of SF, due to the polyanionic characteristics of eADF4(C16). Interactions with counterions result in a decrease of chain repulsion and facilitates molecular interactions.

Typically, the sol-gel transition of silk proteins, either by spontaneous gelling or physical intervention, relates to the self-assembly process [22]. Conformational changes from random coil in sol state to the highly ordered beta sheet structure in gel state were in accordance with the gelation of SF and eADF4(C16). The hydrogen bonding and hydrophobic interaction between hydrophobic glycine-alanine repeats or poly-alanine sequences result in a formation of beta sheet stacks and a subsequent gel formation [5,10]. Our findings showed a reduction of random coil structure as well as an increased beta sheet content after the gelation of all samples (Figure 3B). Notably, the eADF4(C16) showed a lower degree of beta sheet structures than that of SF. As the heavy chain of SF, which mainly directs the formation of beta sheets, possesses a molecular weight of 391 kDa [4], the presence of smaller eADF4(C16) chains with a molecular weight of 47.7 kDa [23] could disturb the molecular organization, resulting in the detected reduction of beta sheet formation upon an increase of eADF4(C16) content.

Cell interactions of SF:eADF4(C16) hydrogels were evaluated using the primary cell, hASC, and two cell lines, L929 mouse fibroblasts and SaOS-2 human osteoblast-like cells. For all cells, those cultured on pure eADF4(C16) hydrogels were rounded, loosely attached to the hydrogel surface, and preferably formed cell aggregates (Figure 5). The surface hydrophilicity, determined from water contact angle measurement of blended films (Figure S1), and the protein adsorptivity of the hydrogels (Figure 3C) could not strongly influence the cellular adhesion. In addition, as the isoelectric point of SF and eADF4(C16) was approximately 3.9 and 3.5, respectively [24,25], the negatively charged materials at the physiological pH (pH 7.4) could not well support cellular attachment through electrostatic interaction with negative-charged cell surface.

The proliferation of hASC primary cells on all hydrogels was slower, and a lower cell number was obtained than that on the TCP control (Figure 4A). SF and eADF4(C16) hydrogels were biocompatible, as seen from no dead cells in all samples (Figure 6). However, the lack of cell adhesion motifs in eADF4(C16), such as RGD, resulted in a low cellular adhesion to the material [26,27]. Furthermore, cells maintained their spherical forms and preferably formed micro-aggregates due to the loose attachment on the hydrogel surface [28]. Therefore, an extended initial lag phase of primary cells with low proliferation potential could be observed, and any growth phase could not be observed within the experimental period.

For L929 fibroblastic cells, proliferation rate depended on the type of samples, as hydrogels with high SF content supported cellular activities better (Figure 4B). Apart from the reason that eADF4(C16) cannot support proper cellular adhesion [12,29], Yamada et al. reported the presence of fibroblast growth-promoting peptides, VITTDSDGNE and NINDFDED, at the N-terminus of SF heavy chain [30]. Hence, the higher content of SF in the blended materials could be beneficial in promoting the proliferation of L929 fibroblasts.

As shown in Figure 4C, the growth of SaOS-2 on 10:0 hydrogels were statistically similar to the control (TCP) on day 5 and 7, and the values were proportionally reduced with increasing eADF4(C16) content. Furthermore, as noticed from Figure 6, the hASC could spread more on hydrogels with higher SF content. It can be presumed that the material acts as a physical cue directing the cellular activities including cytoskeleton organization and cell morphology [31]. Cells cultured on the stiffer materials exhibit a stretch morphology, generating a greater force on actin cytoskeleton, which favor an osteogenic expression [32,33].

The blended hydrogels, especially the 7:3 and 5:5 SF:eADF4(C16) formulations, exhibited accelerated gelation kinetics together with an enhanced cellular activity. Our results showed that the physical and biological properties of the hydrogels are tunable depending on the blending ratio. The simple blending of two different silk proteins reflects a simple preparation route to obtain the hydrogels with required properties such as biological activity of mechanical features for an intended cell biological application.

## 5. Conclusions

Blended SF:eADF4(C16) solutions show accelerated sol-gel transition in combination with the enhanced cell binding of the resulting hydrogels. Faster gelation was noticed with an increment of eADF4(C16) content to at least 50%. At the ratio of 7:3 and 5:5 SF:eADF4(C16), an enhanced cellular adhesion as well as cell proliferation have been noticed. The developed blended hydrogels supported viability of hASC primary cells. Proliferation of cell lines depended on SF content, as an increasing SF content enhanced cell proliferation. The different behavior of each cell type on the blended SF:eADF4(C16) hydrogels could serve as the fundamental data to design the applications of such hydrogels in the future. In addition, for further works, the mechanical properties of the SF:eADF4(C16) should be evaluated, both the modulus and the thixotropic properties of the hydrogels, to investigate their utilization as a printable bioink. Since the high crystallization of SF could limit the shear thinning and self-recovery of the hydrogels, the addition of eADF4(C16) which is known for its thixotropy [11] could ameliorate the printability of the developed hydrogels.

## Data Availability

Data is contained within the article and Supplementary Materials.

## Supplementary Materials

The following are available online at www.mdpi.com/article/10.3390/polym13234182/s1, Figure S1: Water contact angle of blend SF:eADF4(C16) films.
