# Supplementary material for: Impacts of Blended Bombyx mori Silk Fibroin and Recombinant Spider Silk Fibroin Hydrogels on Cell Growth
Source: Polymers (Basel). 2021 Nov 29;13(23):4182. doi: 10.3390/polym13234182 (PMC8659740; doi:10.3390/polym13234182)
Supplement: Supplementary file 1 [file polymers-13-04182-s001.zip › polymers-1459567-supplementary.pdf]

Supplementary information

Manuscript title: Interactions of blended *Bombyx mori* silk fibroin and recombinant spider silk fibroin hydrogels with cells

Authors: Chavee Laomeephol[1], Apichai Vasuratna[2], Juthamas Ratanavaraporn[1,3,4], Sorada Kanokpanont[1,3,5], Jittima Amie Luckanagul[1,6], Martin Humenik[7], Thomas Scheibel[7]*, Siriporn Damrongsakkul[1,3,5]*

Affiliations: [1]Biomaterial Engineering for Medical and Health Research Unit, Faculty of Engineering, Chulalongkorn University, Bangkok 10330, Thailand

[2]Department of Obstetrics and Gynecology, Faculty of Medicine, Chulalongkorn University, Bangkok 10330, Thailand

[3]Biomedical Engineering Research Center, Faculty of Engineering, Chulalongkorn University, Bangkok 10330, Thailand

[4]Biomedical Engineering Program, Faculty of Engineering, Chulalongkorn University, Bangkok 10330, Thailand

[5]Department of Chemical Engineering, Faculty of Engineering, Chulalongkorn University, Bangkok 10330, Thailand

[6]Department of Pharmaceutics and Industrial Pharmacy, Faculty of Pharmaceutical Sciences, Chulalongkorn University, Bangkok 10330, Thailand

[7]Department of Biomaterials, Faculty of Engineering Science, University of Bayreuth, Prof.-Rüdiger-Bormann Str.1, 95447 Bayreuth, Germany

*Corresponding authors

Thomas Scheibel, Email: thomas.scheibel@bm.uni-bayreuth.de, Tel: +49-921-55-6700

Siriporn Damrongsakkul, Email: siriporn.d@chula.ac.th, Tel: +662-218-6862, Fax: +662-218-6877

Materials and Methods

Surface wettability test

Hydrophilicity of the SF:eADF4(C16) blended films was evaluated using a water contact angle tester (DSA 10-MK2, Kruss Scientific, Germany). Thin films of SF:eADF4(C16) were cast onto glass slides and allowed to dry at room temperature for 7 days. After that, water was dropped on the films and the contact angle was measured in quadruplicate. All experiments were performed in triplicate.

Results

Surface wettability of the protein films

Wettability of SF:eADF4(C16) films was analyzed from water contact angles. As presented in **Figure S1**, there was a slight difference between each sample, and the smallest angle was observed for eADF4(C16) films.

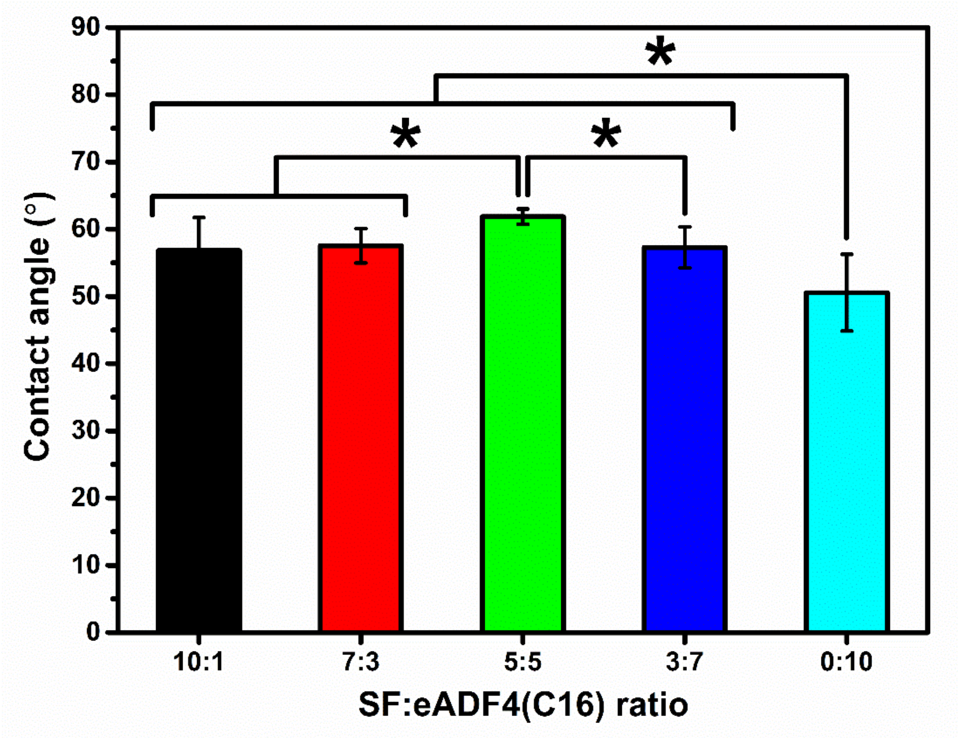

Figure S1 Water contact angle of blend SF:eADF4(C16) films. The asterisk (*) indicates the statistical difference at *p-value* ≤ 0.05.
